# Human papillomavirus prevalence in first, second and third cervical cell samples from women HPV-vaccinated as girls, Denmark, 2017 to 2024: data from the Trial23 cohort study

**DOI:** 10.2807/1560-7917.ES.2025.30.27.2400820

**Published:** 2025-07-10

**Authors:** Mette Hartmann Nonboe, George Maria Napolitano, Jeppe Bennekou Schroll, Berit Andersen, Mary Holten Bennetsen, Sanne Christiansen, Anna Poulsgaard Frandsen, Carsten Rygaard, Rouzbeh Salmani, Estrid Vilma Solyom Høgdall, Elsebeth Lynge

**Affiliations:** 1 Centre for Health Research, Zealand University Hospital, Nykøbing Falster, Denmark; 2 Department of Clinical Medicine, University of Copenhagen, Copenhagen, Denmark; 3 Department of Public Health, University of Copenhagen, Copenhagen, Denmark; 4 Department of Gynaecology and Obstetrics, Herlev Gentofte University Hospital, Herlev, Denmark; 5 Center for Evidence-Based Medicine Odense (CEBMO) and Cochrane Denmark, University of Southern Denmark, Odense, Denmark; 6 UNICCA - University Research Clinic for Cancer Screening, Department of Public Health Programmes, Randers Regional Hospital, Randers, Denmark; 7 Department of Clinical Medicine, Aarhus University, Aarhus, Denmark; 8 Department of Pathology, Randers Regional Hospital, Randers, Denmark; 9 Department of Pathology, Sydvestjysk Hospital, Esbjerg, Denmark; 10 Department of Pathology, Aalborg University Hospital, Aalborg, Denmark; 11 Department of Pathology, Zealand University Hospital, Roskilde, Denmark; 12 Department of Pathology, Herlev Gentofte University Hospital, Herlev, Denmark

**Keywords:** human papillomavirus, prevalence, cervical screening, Denmark, HPV vaccination

## Abstract

**BACKGROUND:**

Danish women vaccinated with the 4-valent human papillomavirus (HPV) vaccine (HPV types: 6/11/16/18) at age 14 in 2008 reached screening age in 2017, allowing assessment of long-term effects on prevalence, persistence and incidence of HPV infections.

**AIM:**

To examine the HPV status of cervical samples over time among women vaccinated as girls.

**METHODS:**

Between February 2017 and February 2024, residual material from cytology-analysed samples collected through the ‘Trial23’ study, part of the national screening programme, was tested for HPV16/18 and non-vaccine high-risk (HR) HPV types. Prevalence in first, second and third samples, and persistence and incidence between samples were calculated.

**RESULTS:**

Over 7 years, 8,659 women provided at least one sample, 5,835 at least two and 2,461 at least three. In 7,800 vaccinated women, HPV16/18 prevalence was 0.4% (95% confidence interval (CI): 0.2–0.5), 0.3% (95% CI: 0.1–0.4) and 0.2% (95% CI: 0.0–0.4) in three consecutive samples. Prevalence of non-vaccine HR HPV was 32% (95% CI: 31–33), 28% (95% CI: 27–29) and 31% (95% CI: 29–33). Persistence of HPV16/18 and non-vaccine HPV among vaccinated women was 40% and 53%. In adjusted analyses comparing vaccinated vs unvaccinated women, incidence was significantly lower for HPV16/18 (adjusted relative risk (aRR) < 0.10) while incidence of non-vaccine HR HPV types was higher (aRR: 1.66; 95% CI: 1.12–2.45). No significant difference was observed for persistence.

**CONCLUSION:**

Our study provides real-world evidence of stable protection against HPV16/18 infections in women vaccinated as girls. Less intensive screening seems reasonable until women vaccinated with the 9-valent vaccine reach screening age, when screening should be reconsidered.

**Key public health message**

**What did you want to address in this study and why?**

Women in Denmark who were vaccinated in 2008 against human papillomavirus (HPV) as girls (~14 years) have reached screening age. Compared with previous generations, these women are expected to have a considerably lower risk of cervical cancer and it is pertinent to assess the new generations’ future need for screening. We tested up to three consecutive cervical cell samples of Danish women (22–30 years) vaccinated as girls, collected between 2017 and 2024.

**What have we learnt from this study?**

Infection with HPV types covered by the vaccine (HPV16/18) has been almost eliminated. Before vaccination, the prevalence of HPV16/18 was between 15–17%, which has decreased in vaccinated women to < 1% by 2021. However, about one-third of women still had HPV infection with non-vaccine high-risk HPV types, and new infections with these types were more frequent in vaccinated than in unvaccinated women.

**What are the implications of your findings for public health?**

The HPV vaccine has been effective in reducing infections with vaccine-covered HPV types (HPV16/18), the HPV types responsible for over 70% of cervical cancer. Still, the high but steady proportion of women infected with high-risk HPV types not covered by the vaccine indicate a need for less intensive but continued screening of these generations.

## Introduction

Persistent infection with human papillomavirus (HPV) poses a high risk of developing cervical cancer and is casually related to other less frequent cancers [1]. Screening and vaccination against HPV can prevent the development of cervical cancer. Most European countries have a long tradition for secondary prevention of cervical cancer by screening for precancerous lesions [2,3]. Previous screening with cytology is now increasingly replaced with HPV-based screening [4,5]. From the end of the 2000s, also primary prevention through vaccination became available. Three prophylactic HPV-vaccines are licensed by the European Medical Agency (EMA) for use in European countries. The 4-valent HPV vaccine covers high-risk (HR) HPV types 16/18 and low-risk types 6/11, the 2-valent covers HPV types 16/18 and the 9-valent covers HPV types 6/11/16/18/31/33/45/52/58. The HR HPV types covered by the 2-valent and 4-valent are responsible for 70% of cervical cancers [6,7], and the HR HPV types covered by the 9-valent vaccine are responsible for up to 90% of cervical cancers [8].

In October 2008, Denmark started to offer free public vaccination with the 4-valent HPV vaccine in a three-dose regime, first inviting all girls aged 13–15 years, and from 1 January 2009, all girls turning 12 years. Coverage with a first dose was 80–90%. The 2-valent vaccine briefly replaced the 4-valent vaccine in 2016 until Denmark switched to the 9-valent vaccine in November 2017. Since September 2019, the vaccination has been gender neutral [9]. Since 2016, the two-dose regime has been used in adolescents under the age of 15 years, while the three-dose regime continues at in those aged 15 and above [10]. Before HPV vaccination started in Denmark, HR HPV was found in all cervical cancers, with 74% from HPV 16/18, and 26% from non-vaccine HR HPV types, i.e. types not covered by the 2- and 4-valent vaccine [11]. 

In Denmark, women are offered cervical screening from age 23 until age 64 years. In 2017, one of the first birth cohorts of women in Denmark who were HPV-vaccinated as teenage girls in 2008 reached the screening age of 23 years. Due to the expected lower cervical cancer risk following vaccination, careful monitoring of screening outcomes in these birth cohorts is needed to reevaluate screening protocols. Trial23, a study part of the national cervical screening programme, was designed to determine the prevalence of HPV infection in cervical cell samples over three rounds of screening invitations. The Trial23 population has now been followed up for 7 years, providing the unique possibility to evaluate the effect of the vaccine over consecutive cervical cell samples. In a previous study, we reported the number of follow-up screenings required to detect one case of severe cervical precancerous lesions only after the first screening [12]. Here, we report the HPV prevalence in three consecutive samples, and on the changes in HPV persistence and incidence between the first and second samples, and the second and third samples.

## Methods

### Trial23 study design

Trial23 was designed as a public health study embedded in the Danish national cervical screening programme. The study design has been reported previously [12-14]. In brief, the study population included women born in 1994, who turned 23 in 2017 and were living in Denmark on 1 January 2017. The women were all offered HPV vaccination with the 4-valent vaccine (Gardasil, Merck & Co., Inc.) from October 2008, when most of them had turned 14 years old. In Denmark, women are invited for their first screening at age 23, hence the name Trial23. Half of the women were randomised to have the residual material of their cervical cell samples HPV-tested.

In Denmark, all cervical cell samples are analysed in one of six pathology departments. In the present study, four of these departments participated, representing women from Region North (Aalborg pathology department), Central Region (Randers pathology department), Region Zealand (Næstved pathology department) and Region South (municipalities covered by Esbjerg pathology department), equating to ⁓55% of the population of women aged 23–64 years in Denmark. The municipality codes for the Trial23 study area are provided in Supplementary Table S1. The randomisation was linked to the woman’s personal identification number, and the allocation was visible when an incoming cell sample was scanned at the participating pathology departments. According to the allocation, received samples were coded as (i) cytology with additional HPV testing (HPV-arm, code ‘T23J’), or as (ii) cytology only (cytology arm, ‘T23N’). More detailed codes are listed in Supplementary Table S2 and Supplementary Figure S1. Here, we report only on data from the HPV arm.

All cell samples received between 1 February 2017 and 29 February 2024 were included in the study, allowing for up to three screening invitations per woman. Although some women had provided up to 10 cell samples within the study period, we only included the first three samples in our analysis. In the analysis, we included only cell samples taken at least 3 months apart, according to Danish recommendations.

### Human papillomavirus testing

Standard SurePath liquid-based medium (Becton, Dickinson and Company, United States) procedures were used. Cell samples were first used for cytology analysis with results coded according to Bethesda classification (the M-codes to cytology diagnosis are provided in Supplementary Table S3). The HPV testing was performed on the residual material with the Cobas 4800/6800 HPV DNA test (Roche Diagnostics, Switzerland). The Cobas system has four test-result channels: +/− HPV16; +/− HPV18; +/− for non-vaccine HR HPV types (31, 33, 35, 39, 45, 51, 52, 56, 58, 59, 66 and 68), and a control channel. In April 2023, one pathology department, switched to Seegene Starlets Allplex HPV28, which was comparable with Roche Cobas [15]. One percent of the cell samples included where analysed with Seegene. 

### Data linkage

Personal identification numbers (PIN) and dates of death, migration and emigration were retrieved from the Danish Civil Registration System. Data on cytology cell samples were provided by the participating pathology departments and supplemented with the National Pathology Register. The HPV test results were retrieved from the participating pathology departments. 

Data on HPV vaccination were retrieved from the Health Insurance Register, supplemented with registrations of self-paid vaccines from the Drug Prescription Register and linked using the personal identification numbers. We considered a woman vaccinated if she had at least one dose of HPV vaccine registered, independent of age at vaccination. Women were not screened prior to vaccination. See Supplementary Table S4 for an overview of the codes used from the Danish National Health Registers.

### Statistical analysis

We estimated the cumulative screening coverage with one, two or three cell samples among the randomised women from the Trial23 study area over the 7 years of data collection. Since we did not have access to the population data from the randomisation module, and some women had moved in and out of the Trial23 study area during the period, the coverage was estimated based on data from the Danish Civil Registration System and the National Pathology Register. For all years, we defined the denominator as number of randomised women having lived in the Trial23 study area at any time during the data collection period. For each year, the numerator was defined as number of women in the denominator with at least one cell sample taken in Denmark between the beginning of data collection and that year. Similar calculations were made for cumulative coverage with at least two or three cell samples.

Our primary outcome was the HPV prevalence in first, second and third cell sample of the subgroups detectable by Cobas 4800/6800: (i) positive for at least one of the 14 HR HPV types (16, 18, 31, 33, 35, 39, 45, 51, 52, 56, 58, 59, 66 and 68); (ii) positive for either HPV16, HPV18 or both; (iii) positive for any of the 12 non-vaccine HR HPV types; and (iv) HR HPV-negative. To distinguish between old (‘persistent’) and new (‘incident’) infections, we further stratified the prevalence at second and third samples by previous infection status. Prevalence estimates were reported with 95% Clopper–Pearson confidence intervals (CI).

We compared prevalence, persistence and incidence among vaccinated and unvaccinated women. The comparison was made on the multiplicative scale by estimating relative risks obtained through Poisson regression models using a sandwich variance estimator. Two models are reported: Model 1 as a crude model (no adjustment); Model 2 with adjustments for age at cell sampling (natural cubic spline with 2 degrees of freedom), pathology department (categorical, four levels) where the sample was analysed, and, for persistence and incidence proportions, further adjusted for pathology department where the previous sample was analysed, and time (continuous) since previous testing. Counts less than three are not reported, in accordance with local rules for data security.

All data manipulation, statistical analyses and plots were done using R software version 4.4.1, and software packages tidyverse [16], sandwich [17,18] and lmtest [19].

## Results

**Figure 1:**
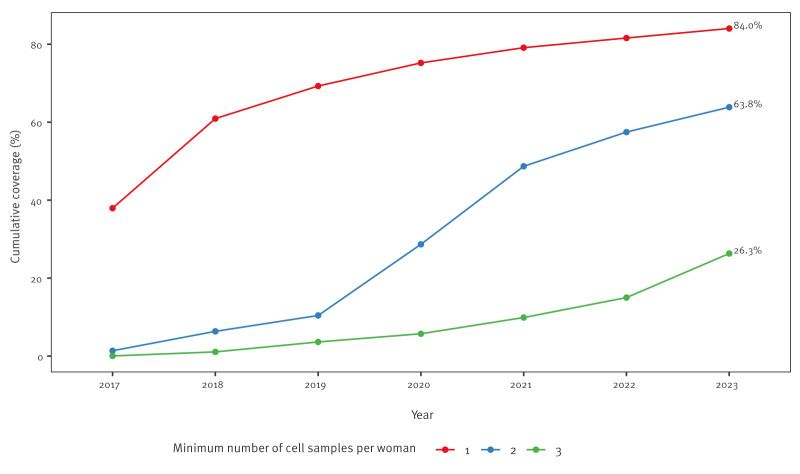
Cumulative sampling coverage of women living in the Trial23 study area at least once during the data collection period, Denmark, 1 February 2017–29 February 2024 (n = 23,202)

In total, 23,202 women lived in the study area at least one time during part of the study period. Of these, 84% (n = 19,495) had at least one cell sample during the study period registered in the National Pathology Register, and 16% (n = 3,707) had no cell sample. The cumulative screening coverage with at least one cell sample increased from 38% (n = 8,800) in 2017 to 61% (n = 14,132) in 2018; whereafter there was a steady increase up to the 84% (n = 19,495) by the end of 2023 (Figure 1). By the end of the observation period, the cumulative coverage with at least two and three cell samples was 64% and 26%, respectively. 

The denominator (n = 23,202) represents women born in 1994 and living in Denmark on 1 January 2017, who have had their address registered in the study area at least once between 1 February 2017 and 29 February 2024 (for simplicity here marked on the X-axis from 2017 to 2023). The numerators for each data point represent the cumulative number of women in the denominator who have had at least one cervical cell sample examined in any pathology department in Denmark between 2017 and the specified year, 2017, 2018, […], 2023. Note that from 2017 to 2023, the women aged from 23 to 29 years. Cell samples were taken at least 3 months apart.

### Women included in Trial23

In the data provided by the participating pathology departments, 17,252 women with at least one cervical cell sample were registered between 1 February 2017 and 29 February 2024. Supplementary Figure S1 provides a flowchart with numbers of women randomised to both cytology and HPV testing of residual material, or to cytology only. After excluding 29 women with missing/ambiguous diagnoses and 1,270 cell samples collected as cell sample numbers 4–10, this analysis of data from the HPV arm included a total of 8,659 women, and 16,955 cell samples taken as cell samples 1–3.

The mean age of women at first cell sample was 24.4 years, with 62% aged 22–23 (Table 1), at second cell sample, 26.6 years, with 59% aged 26–27, and at third cell sample 27.7 years, with 53% aged 28 or older. Of the women with at least one cell sample, 90% were HPV-vaccinated; see Supplementary Table S5 for data by vaccination status.

**Table 1:** Characteristics of women in the HPV arm of Trial23 at first, second and third cell sample, Denmark, 1 February 2017–29 February 2024 (n = 8,659 women)

Characteristics	Cell sample 1		Cell sample 2		Cell sample 3	
	Number of women	%	Number of women	%	Number of women	%
Total	8,659	100	5,835	100	2,461	100
**Age in years at sample collection**						
22–23	5,335	62	425	7	24	1.0
24–25	1,632	19	888	15	465	19
26–27	1,152	13	3,453	59	660	27
≥ 28	540	6	1,069	18	1,312	53
Mean age (SD)	24.4 (1.7)		26.6 (1.5)		27.7 (1.7)	
**Years of collection**						
2017–18	5,906	68	664	11	113	4.6
2019–20	1,512	18	1,886	32	472	19
2021–22	845	10	2,555	44	789	32
2023–24	396	4.6	730	13	1,087	44
**HPV vaccination status**						
No	859	10	437	8	173	7
Yes	7,800	90	5,398	93	2,288	93
Mean age at vaccination (SD)	14.4 (0.5)		14.4 (0.5)		14.5 (0.5)	
**Cytology**						
NILM	7,677	89	5,153	88.3	2,090	85
ASCUS	445	5	276	4.7	152	6
LSIL	351	4.1	236	4	108	4.4
ASCH/AGS/AIS	40	0.5	45	0.8	35	1.4
HSIL	70	0.8	55	0.9	29	1.2
Unsatisfactory/other	76	0.9	70	1.2	47	1.9

AGC: atypical glandular cells; AIS: adenocarcinoma in situ; ASCH: atypical squamous cells cannot exclude HSIL; ASCUS: atypical squamous cells of undetermined significance; HPV: human papillomavirus; HSIL: high grade squamous intraepithelial lesion; LSIL: low grade squamous intraepithelial lesion; NILM: negative for intraepithelial lesion or malignancy; SD: standard deviation.

### Human papillomavirus prevalence

Among the 8,659 women with a first cell sample, the prevalence of any HR HPV infection was 32% (95% confidence interval (CI): 31–33) (Table 2). Among women with a second (n = 5,835) and third (n = 2,461) cell sample, HPV prevalence was 28% (95% CI: 27–29) and 31% (95% CI: 29–33), respectively. The prevalence of HPV16/18 types was low and stayed stable over time, at 1.0% 95% CI: 0.8–1.2) in first samples, 0.6% (95% CI: 0.4–0.9) in second, and 0.6% (95% CI: 0.3–1.0) third samples. The prevalence of all HPV infections resembles the prevalence of non-vaccine HR HPV, given the very low prevalence of HPV16/18.

**Table 2:** Prevalence of HPV in first, second and third cell samples, Denmark, 1 February 2017–29 February 2024 (n = 8,659 women)

HPV diagnosis	Cell sample 1^a^			Cell sample 2^b^			Cell sample 3^c^		
	n	Prevalence		n	Prevalence		n	Prevalence	
		%	95% CI		%	95% CI		%	95% CI
Positive for at least one of the 14 HR HPV types^d^	2,789	32	31–33	1,612	28	27–29	756	31	29–33
Positive for either HPV16, HPV18 or both^e^	83	1.0	0.8–1.2	37	0.6	0.4–0.9	14	0.6	0.3–1.0
Positive for any of the 12 non-vaccine HR HPV types^e,f^	2,737	32	31–33	1,590	27	26–28	745	30	29–32
HPV-negative	5,870	68	67–69	4,223	72	71–734	1,705	69	67–71
Total	8,659	NA		5,835	NA		2,461	NA	

CI: confidence interval; HPV: human papillomavirus; HR: high-risk; NA: not applicable.

^a^ Any first cervical cell sample, independent of date.

^b^ Any second cervical cell sample, at least 3 months after the first cell sample.

^c^ Any third cervical cell sample, at least 3 months after the second cell sample.

^d^ At least one of the HR HPV types: 16, 18, 31, 33, 35, 39, 45, 51, 52, 56, 58, 59, 66 and 68.

^e^ Results are overlapping, as a sample can be positive for both HPV16/18 and non-vaccine HR HPV types because of coinfections.

^f^ At least one of the non-vaccine HR HPV types: 31, 33, 35, 39, 45, 51, 52, 56, 58, 59, 66 and 68.

**Figure 2:**
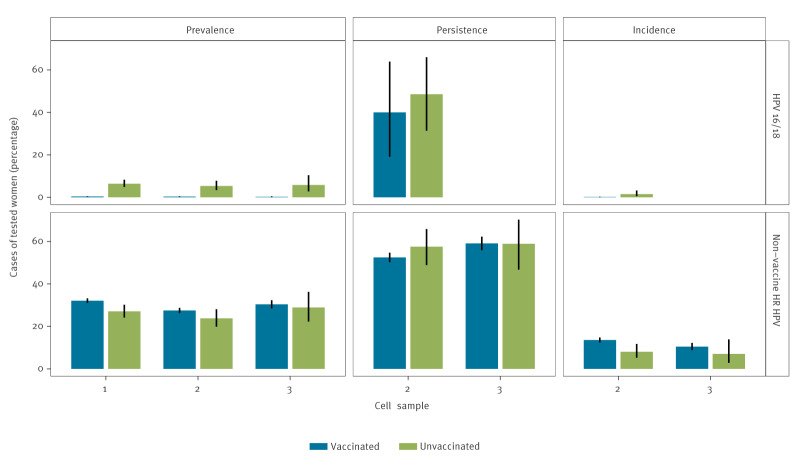
Prevalence, persistence and incidence of HPV16/18 and non-vaccine high-risk HPV infections in HPV-vaccinated (n =7,800) and unvaccinated women (n = 859) in first, second and third cervical cell samples, Denmark, 1 February 2017–29 February 2024

HPV: human papillomavirus; HR: high-risk.

Among the 7,800 vaccinated women, HPV16/18-prevalence was 0.4% (95% CI: 0.2–0.5) at the first cell sample, 0.3% (95% CI: 0.1–0.4) at the second cell sample and 0.2% (95% CI: 0.0–0.4) at the third cell sample, and the prevalence of non-vaccine HR HPV types was 32% (95% CI: 31–33), 28% (95% CI: 27–29) and 30% (95% CI: 27–29), respectively (Figure 2; see Supplementary Table S6 for absolute numbers). Among the 859 unvaccinated women, HPV16/18 prevalence was 6%, 5%, and 6%, and non-vaccine HR HPV was 27%, 24%, and 29%, respectively. The HPV16/18 prevalence was statistically significantly lower in vaccinated than in unvaccinated women, the adjusted relative risk (aRR) for HPV16/18 at the first sample was 0.06 (95% CI: 0.04–0.09), at second sample 0.05 (95% CI: 03–0.10), and at the third sample 0.04 (95% CI: 0.01–0.11) (Table 3). The prevalence of non-vaccine HR HPV types was not significantly different in vaccinated than in unvaccinated women, with aRR 1.11 (95% CI: 0.99–1.24), 1.14 (95% CI: 0.97–1.34) and 1.12 (95% CI: 0.89–1.14) at first, second and third sample, respectively, but all 95% CIs included 1.

The X-axis indicates the number of cell samples (1–3). The Y-axis shows the percentage of cases positive for either HPV16/18 or for non-vaccine HR HPV types in each sample.

**Table 3:** Regression models of relative risk of prevalence, persistence and incidence, in HPV-vaccinated (n = 7,800) vs unvaccinated women (n = 859), Denmark, 1 February 2017–29 February 2024

Outcomes	Vaccinated vs unvaccinated							
	HPV16/18				Non-vaccine HR HPV			
	Model 1^a^		Model 2^b^		Model 1^a^		Model 2^b^	
	RR	95% CI	aRR	95% CI	RR	95% CI	aRR	95% CI
**Prevalence**								
Cell sample 1	0.06	0.04–0.09	0.06	0.04–0.09	1.18	1.06–1.33	1.11	0.99–1.24
Cell sample 2	0.05	0.03–0.10	0.05	0.03–0.10	1.16	0.97–1.38	1.14	0.97–1.34
Cell sample 3	0.03	0.01–0.10	0.04	0.01–0.11	1.05	0.83–1.34	1.12	0.89–1.41
**Persistence**								
Cell sample 2	0.82	0.44–1.56	0.81	0.43–1.55	0.91	0.79–1.06	0.95	0.83–1.10
Cell sample 3	NA		NA		1.00	0.82–1.22	1.07	0.88–1.30
**Incidence**								
Cell sample 2	0.07	0.02–0.23	0.08	0.02–0.23	1.68	1.14–2.49	1.66	1.12–2.45
Cell sample 3	NA		NA		1.49	0.72–3.10	1.58	0.76–3.28

aRR: adjusted relative risk; CI: confidence interval; HPV: human papillomavirus; HR: high-risk; NA: not applicable; RR: relative risk.

^a^ Crude model.

^b^ Model adjusted for age at cell sampling, pathology department where the cell sample was analysed; for persistence and incidence further adjusted for pathology department where previous cell sample was analysed and time since previous HPV test. Data by age group presented in Supplementary Figure S3.

Persistence and incidence can only be reported between first and second sample (called cell sample 2) and between second and third cell sample (called cell sample 3), as this is calculated based on the results of the previous sample.

### Human papillomavirus persistence and incidence

Of the 83 women with a first sample positive for HPV16/18, 55 were retested, and of these, 25 had an HPV16/18-positive second sample, giving a persistence of 45% (Table 4). Similarly, of the 37 women with a second cell sample positive for HPV16/18, 22 were retested, and the third cell sample was HPV16/18-positive for 11 women, giving a persistence of 50%. Of the 8,576 women with a first cell sample negative for HPV16/18, 5,780 were retested, and 12 second cell samples were HPV16/18-positive, giving an incidence of 0.2%. Of 5,798 women with a second cell sample negative for HPV16/18, 2,439 were retested, and three had a third cell sample positive for HPV16/18, giving an incidence of 0.1%. For non-vaccine HR HPV, persistence was 53% from the first to the second cell sample, and 59% from the second to third cell sample, with corresponding incidence of 13% and 10%, respectively.

**Table 4:** Persistent and incident HPV infections in the total of retested women between first and second, and between second and third cell samples, Denmark, 1 February 2017–29 February 2024 (n = 8,659)

HPV type	Consecutive sample			
HPV16/18				
**Cell sample 1**	**Cell sample 2**			**Total**
	**HPV16/18**	**No HPV16/18**	**No test**	
HPV16/18	25	30	28	83
No HPV16/18	12	5,768	2,796	8,576
Total	37	5,798	2,824	8,659
Persistence (HPV16/18)	45%			
Incidence (HPV16/18)	0.2%			
**Cell sample 2**	**Cell sample 3**			**Total**
	**HPV16/18**	**No HPV16/18**	**No test**	
HPV16/18	11	11	15	37
No HPV16/18	3	2,436	3,359	5,798
Total	14	2,447	3,374	5,835
Persistence (HPV16/18)	50%			
Incidence (HPV16/18)	0.1%			
Non-vaccine HR HPV types				
**Cell sample 1**	**Cell sample 2**			**Total**
	**Non-vaccine HR HPV**	**No non-vaccine HR HPV**	**No test**	
Non-vaccine HR HPV	1,097	980	660	2,737
No non-vaccine HR HPV	493	3,265	2,164	5,922
Total	1,590	4,245	2,824	8,659
Persistence (HPV other)	53%			
Incidence (HPV other)	13%			
**Cell sample 2**	**Cell sample 3**			**Total**
	**Non-vaccine HR HPV**	**No non-vaccine HR HPV**	**No test**	
Non-vaccine HR HPV	597	413	580	1,590
No non-vaccine HR HPV	148	1,303	2,794	4,245
Total	745	1,716	3,374	5,835
Persistence (HPV other)	59%			
Incidence (HPV other)	10%			

HR: high-risk; HPV: human papillomavirus.

Non-vaccine HR HPV: 31, 33, 35, 39, 45, 51, 52, 56, 58, 59, 66 and 68. Absolute numbers stratified by vaccination status can be found in Supplementary Table S7.

**Figure 3:**
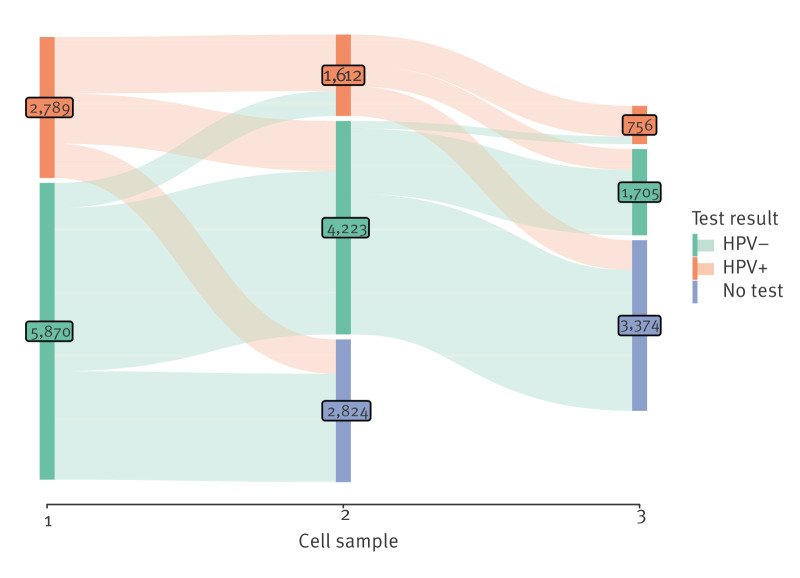
Sankey graph of change in HPV test results at first, second and third cell sample over time, Denmark, 1 February 2017–29 February 2024 (n = 8,659)

In vaccinated, retested women, persistence of HPV16/18 between first and second cell samples was 40%, and in unvaccinated women it was 49%, (aRR: 0.81; 95% CI: 0.43–1.55) (Table 3 and 4, Figure 2). The results spilt by vaccination status are provided in Supplementary Table S7. The incidence of HPV16/18 was 0.1% in vaccinated vs 1.5% in unvaccinated women, (aRR: 0.08; 95% CI: 0.02–0.23) (Table 3, Figure 2). In vaccinated women, persistence of non-vaccine HR HPV between first and second cell sample was 53%, between second and third 59%, and in unvaccinated women, the numbers were 49% and 58%, respectively with aRR: 0.95; (95% CI: 0.83–1.10) and 1.07 (95% CI: 0.88–1.30). In vaccinated, retested women, the incidence of non-vaccine HR HPV was 14% between first and second cell sample, and 10% between second and third, and in unvaccinated women the numbers were 8% and 7%, respectively, with aRR 1.66 (95% CI: 1.12–2.45) and 1.58 (95% CI: 0.76–3.28). For changes in positive/negative status by HPV test result in first cell sample, see Figure 3.

HPV: human papillomavirus.

HPV infection flow charts are provided in Supplementary Figures 2a and 2b, where numbers are shown based on vaccination status.

## Discussion

To our knowledge, this is the first study to report on HPV status in consecutive rounds of cervical cell sampling in a cohort of young women offered 4-valent HPV vaccination as girls. Over the 7 years of data collection, 84% of women randomised in the Trial23 study area had at least one cell sample taken. In the routine statistics on cervical screening in Denmark, coverage is calculated for women aged 27–29 years as the percentage of women in the population register on a given date with at least one cell sample within the last 3.5 years [20]. For years 2018–22, this percentage was 72% for the Trial23 study area, and thus very close to the coverage in 2019–20 in our data, showing consistency between our data and population data despite different calculation methods. The number of second cell samples in our study peaked in 2021–22, probably because of a delay related to the COVID-19 pandemic [21].

Our data from three rounds of consecutive cell sampling among vaccinated women show that HPV16/18 has been almost eliminated over the 7-year study period. The HPV16/18 prevalence in unvaccinated women remained at a level of 5%, much lower than the 17–19% reported for young Danish women in the pre-vaccination period [22], which strongly indicates population immunity. The prevalence of non-vaccine HR HPV types remained at ⁓30% for vaccinated women and slightly lower (⁓27%) for those unvaccinated, a non-significant difference. Persistence was high for vaccine (~45%) and non-vaccine HPV types (~55%) and independent of vaccination status. Incidence of HPV16/18 was very low in vaccinated women and significantly lower than in unvaccinated women. Remarkably, the incidence of non-vaccine HR HPV types was statistically significantly higher in vaccinated than in unvaccinated women.

In the Danish School Health Survey, 24% of girls born in 1994 reported to have been sexually active at the age of 14 [23], and girls were not HPV-tested before vaccination. We were, therefore, not able to determine whether the few HPV16/18 infections in first cell sample in vaccinated women were present already at the time of vaccination or were breakthrough infections. In any case, the level of persistence of HPV16/18 infections with no difference between vaccinated and unvaccinated women supported the conclusion that the 4-valent HPV-vaccine is not a therapeutic vaccine [24].

In Trial23 data from the first 2 years of data collection, the prevalence of non-vaccine HR HPV types was 34% [25], compared with 36–39% in the pre-vaccination cohorts [11,22]. When including Trial23 data from all 7 years of data collection, the prevalence of non-vaccine HR HPV types in the first cell samples was 32%. This comparison with external data may suggest a possible indication of cross-protection; however, given that the cohorts were not directly comparable, such an interpretation should be viewed with caution. Cross-protection was not indicated in the internal comparison between vaccinated and unvaccinated women. Across age groups, the prevalence of non-vaccine HR HPV types was slightly higher in vaccinated than in unvaccinated women. Furthermore, the incidence of non-vaccine HR HPV infections was significantly higher in vaccinated than in unvaccinated women, though at a low level. These observations could point to type replacement or unmasking [24]. 

The observations from Trial23 are supported by data from Australia [26-28], the English primary HPV-screening pilot [29], the English monitoring system based on samples collected for *Chlamydia trachomatis* testing [30] and from a study embedded in the routine screening from Scotland [31]. A more in-depth comparison of our findings with these studies can be found in the Supplement.

Given that our study showed a very low prevalence of HPV16/18, which remained stable over three rounds of cell sampling, the question arises of whether Denmark should stop screening birth cohorts of women HPV-vaccinated as girls [32]. Here, it should be considered that the prevalence of non-vaccine HR HPV types remained at 30%. In the follow-up based on the routine cytology of women recruited to Trial23 during the first 2 years of data collection, 2.6% were diagnosed with cervical intraepithelial neoplasia stage 2 and above (CIN2 +) [12]. It is reasonable to argue that, nationwide, the detection of CIN2 + related to infections with non-vaccine HR HPV types must have contributed to the presently very low incidence of cervical cancer of 3 per 100,000 in the HPV-vaccinated birth cohorts [33]. However, with the disappearance of HPV16/18-related CIN2 +, resources needed for the detection of remaining CIN2 + cases would increase. 

Due to low vaccination coverage and unclear vaccination records in the United States, Kim et al. argued against adjusting cervical screening strategies based on HPV-vaccination status [34]. These concerns are less relevant in Denmark with high-quality registers [35]. Furthermore, vaccination uptake has been high in Denmark [9]. Determinants of low uptake have included the mother’s non-participation in cervical screening [36], being an immigrant or descendant of an immigrant [37] living without parents or attending special needs education [38]. Despite the general vaccination coverage, this indicator should be closely monitored, as Denmark in 2015 had a considerable dip in coverage due to concern about vaccine side-effects [39], where information from social media played an important role [40]. The strength of our study was its direct integration into the Danish cervical screening programme, providing real-world data of women with cell samples. To avoid a selection bias, we included all cell samples regardless of whether the samples were taken for screening, follow-up of abnormal findings or for control of treatment.

Our study had some limitations. Firstly, not all pathology departments in Denmark participated in the study because of lack of capacity at the time of study initiation. However, the participating departments covered over 50% of all women in the birth cohort and power calculations made before initiation supported the validation of the results . Secondly, vaccine HPV types 6/11 were not assessed. However, these types are not high-risk and not included in the HPV test used in the study. Thirdly, we compared data from vaccinated and unvaccinated women, and we adjusted for age and time intervals between tests. However, we did not have data on socioeconomic status and ethnicity, and we could not exclude a selection bias in, for instance, sexual activity. Fourthly, the estimates for persistence and incidence were obtained only from retested women, and they may potentially differ from non-retested women. Finally, it should be stressed that our results cannot be interpreted as showing the effect of primary HPV screening.

## Conclusion

We analysed the presence of HPV infections in up to three consecutive cell samples from women aged 22–30 and vaccinated with the 4-valent HPV-vaccine as girls. Our study provided solid, real-world evidence of stable protection against HPV16/18. A high proportion of the few HPV16/18-infections in vaccinated women remained persistent. Across the three rounds of cell sampling, the prevalence of non-vaccine HR HPV infections remained high in both vaccinated and unvaccinated women, while the incidence of non-vaccine HR HPV types was significantly higher in vaccinated than in unvaccinated women. Therefore, less intensive screening seems reasonable until women vaccinated as girls with the 9-valent vaccine reach screening age, at which point screening should be reconsidered.

## Data Availability

The data underlying the results of this study have been managed in compliance with EU data protection regulations and all pertinent data are included in the paper. Data are stored in an approved research data repository within Statistics Denmark. Researchers seeking access to a pseudonymised version of the data for projects that offer societal benefits may be granted access, provided they meet the criteria for handling confidential data as outlined by EU regulations. Detailed information on how to apply can be found at: https://english.sundhedsdatastyrelsen.dk/health-data-and-registers/research-services/apply-for-data, or by contacting forskerservice@dst.dk and menon@regionsjaelland.dk

## Supplementary Data

1048000 Supplement
