# Supplementary material for: Human papillomavirus prevalence in first, second and third cervical cell samples from women HPV-vaccinated as girls, Denmark, 2017 to 2024: data from the Trial23 cohort study
Source: Euro Surveill. 2025 Jul 10;30(27):2400820. doi: 10.2807/1560-7917.ES.2025.30.27.2400820 (PMC12262111; doi:10.2807/1560-7917.ES.2025.30.27.2400820)
Supplement: Supplement [file 24-00820_NONBOE_Supplement.pdf]

SUPPLEMENTARY MATERIAL

This supplementary material is hosted by *Eurosurveillance* as supporting information alongside the article Human papillomavirus prevalence in first, second and third cervical cell samples from women HPV-vaccinated as girls, Denmark, 2017 to 2024: data from the Trial23 cohort study, on behalf of the authors, who remain responsible for the accuracy and appropriateness of the content. The same standards for ethics, copyright, attributions and permissions as for the article apply. Supplements are not edited by *Eurosurveillance* and the journal is not responsible for the maintenance of any links or email addresses provided therein.

Supplementary Table S1. Municipality codes for the Trial23 study area

Code	Municipality	Region
253	Greve	Zealand
259	Køge	Zealand
265	Roskilde	Zealand
269	Solrød	Zealand
306	Odsherred	Zealand
316	Holbæk	Zealand
320	Faxe	Zealand
326	Kalundborg	Zealand
329	Ringsted	Zealand
330	Slagelse	Zealand
336	Stevns	Zealand
340	Sorø	Zealand
350	Lejre	Zealand
360	Lolland	Zealand
370	Næstved	Zealand
376	Guldborgsund	Zealand
390	Vordingborg	Zealand
510	Haderslev	South
530	Billund	South
540	Sønderborg	South
550	Tønder	South
561	Esbjerg	South
563	Fanø	South
573	Varde	South
575	Vejen	South
580	Aabenraa	South
615	Horsens	Central
657	Herning	Central
661	Holstebro	Central
665	Lemvig	Central
671	Struer	Central
706	Syddjurs	Central
707	Norddjurs	Central
710	Favrskov	Central
727	Odder	Central
730	Randers	Central

740	Silkeborg	Central
741	Samsø	Central
746	Skanderborg	Central
751	Aarhus	Central
756	Ikast-Brande	Central
760	Ringkøbing-Skjern	Central
766	Hedensted	Central
773	Morsø	North
779	Skive	Central
787	Thisted	North
791	Viborg	Central
810	Brønderslev	North
813	Frederikshavn	North
820	Vesthimmerland	North
825	Læsø	North
840	Rebild	North
846	Mariagerfjord	North
849	Jammerbugt	North
851	Aalborg	North
860	Hjørring	North

Supplementary Table S2. Project codes used by participating pathology departments and coding inaccuracy editing

Description	TRIAL23 Codes	Variations of codes sometimes used by the pathology departments	Standard codes
**Project code**	PYYT23	Only T23J or T23N	NA
**Cytology- + HPV-testing**	T23J	t23j<br>23j	NA
**Cytology-testing only**	T23N	t23n<br>23n	NA
**HPV negative**	PYY500	NA	FY5005, FY5001
**HPV positive**	PYY006, PYY45P	NA	FY5006
**Non-vaccine HR HPV**	PYY400, PYY40A	Neg., Oth.<br>Neg., Pos. Oth.	FY5009, FY50010, Æ33400, Æ3340A, Æ3340B, Æ3340C, Æ33431, Æ33433, Æ33435, Æ33439, Æ33445, Æ33451, Æ33452, Æ33456, Æ33458, Æ33459, Æ33466, Æ33468,
**HPV16**	PYY416	Neg., 16	Æ33416
**HPV18**	PYY418	NA	Æ33418

Supplementary Table S3. M-codes to cytology diagnosis

Diagnosis	SNOMED codes
**Unsatisfactory**	M09000 M09010 M09011 M09012 M09013 M09014 M09015 M09016 M09017 M09018 M09019 M0901X M0901Y M09070 M09100 M09140 M09145 M09150 M30610 M37000 M54310
**Normal**	M00100 M00120 M00121 M00122 M01111 M02561 M09450 M09460 M09462 M09463 M11600 M11610 M51620 M69520 M69780 M69784 M69810 M69820 M69880 M74030 MYY122 M4**** M58*** M72*** M73*** (excluding M73005 M73225 M73229 M73309)
**ASCUS**	M67014 M69700 M69711 M72125 M73005 M73225
**LSIL**	M67016 M69701 M69790 M76700 M76701 M76720 M74A*9
**ASCH/AGS/AIS**	M67010 M67020 M69712 M69762
**HSIL**	M67017 M69702 M69703 M69760 M73229 M73309 M74HG9 M80102 M80702 M80722 M80732 M80762 M80812 M81402 M740*9 M74B*9 M74C*9
**Other**	All other M-codes

Supplementary Table S4. Overview of codes used from Danish National health registers

**Codes from the National Register of Pathology (NRP) for cervical screening outcomes[a]**	
Cervical histology	T83* (cervix uteri), T820* (uterus), T829* (corpus and cervix uteri)
Cervical cytology	T8X3* (cervical cytology)
**Codes from the Danish National Prescription Register for HPV vaccination[b]**	
Quadrivalent HPV vaccine (4-valent)	J07BM01
Bivalent HPV vaccine (2-valent)	J07BM02
**Codes from the National Health Services Register (NHSR) for HPV vaccination[c]**	
HPV vaccine 1st dose	808328
HPV vaccine 2nd dose	808329
HPV vaccine 3rd dose	808330

Supplementary Table S5. Characteristics of women in the HPV arm of Trial23 at first, second and third cell sample, Denmark, 1 February 2017–29 February 2024 (n = 8,659) by vaccination status (n = 7800 vaccinated women, n = 859 unvaccinated women)

Vaccinated women						
Characteristics	Cell sample 1		Cell sample 2		Cell sample 3	
	n	%	n	%	n	%
**Number of women**	7800	100%	5398	100%	2288	100%
**Age in years at sample collection**						
22–23	4904	63	394	7.3	21	0.9
24–25	1461	19	805	15	426	19
26–27	980	13	3233	60	611	27
≥ 28	455	5.8	966	18	1230	54
Mean age (SD)	24.3 (1.7)		26.6 (1.5)		27.7 (1.7)	
**Cytology**						
NILM	6,918	89	4,796	89	1,947	85
ASCUS	407	5.2	249	4.6	142	6.2
LSIL	319	4.1	210	3.9	102	4.5
ASCH/AGS/AIS	34	0.4	37	0.7	31	1.4
HSIL	54	0.7	44	0.8	24	1
Unsatisfactory/other	68	0.9	62	1.1	42	1.8
**Unvaccinated women**						
Characteristics	Cell sample 1		Cell sample 2		Cell sample 3	
	n	%	n	%	n	%
**Number of women**	859	100%	437	100%	173	100%
**Age in years at sample collection**						
22–23	431	50	31	7.1	3	1.7
24–25	171	20	83	19	39	23
26–27	172	20	220	50.3	49	28
≥ 28	85	9.9	103	24	82	47
Mean age (SD)	24.9 (2.0)		26.7 (1.6)		27.5 (1.8)	
**Cytology**						
NILM	759	88	357	82	143	83
ASCUS	38	4.4	27	6.2	10	5.8
LSIL	32	3.7	26	5.9	6	3.5
ASCH/AGS/AIS	6	0.7	8	1.8	4	2.3
HSIL	16	1.9	11	2.5	5	2.9
Unsatisfactory/other	8	0.9	8	1.8	5	2.9

Supplementary Table S6. Prevalence of HPV in first, second and third cell samples, Denmark, 1 February 2017–29 February 2024 (n = 8,659) by vaccination status (n = 7800 vaccinated women, n = 859 unvaccinated women)

**Vaccinated women**									
**HPV diagnosis**	**Cell sample 1[a]**			**Cell sample 2[b]**			**Cell sample 3[c]**		
	**n**	**Prevalence**		**n**	**Prevalence**		**n**	**Prevalence**	
		**%**	**95% CI**		**%**	**95% CI**		**%**	**95% CI**
Positive for at least one of the 14 HR HPV types[d]	2521	32.3	31.3-33.4	1,495	27.7	26.5-28.9	699	30.6	28.7-32.5
Positive for either HPV16, HPV18 or both[e]	28	0.4	0.2-0.5	14	0.3	0.1-0.4	4	0.2	0-0.4
Positive for any of the 12 non-vaccine HR HPV types[e,f]	2504	32.1	31.1-33.2	1,486	27.5	26.3-28.7	659	30.4	28.5-32.3
HPV-negative	5279	67.7	66.6-68.7	3,903	72.3	71.1-73.5	1,589	69.4	67.5-71.3
Total	7800	NA		5,398	NA		2,288	NA	
**Unvaccinated women**									
**HPV diagnosis**	**Cell sample 1[a]**			**Cell sample 2[b]**			**Cell sample 3[c]**		
	**n**	**Prevalence**		**n**	**Prevalence**		**n**	**Prevalence**	
		**%**	**95% CI**		**%**	**95% CI**		**%**	**95% CI**
Positive for at least one of the 14 HR HPV types[d]	268	31.2	28.1-34.4	117	26.8	22.7-31.2	57	32.9	26-40.5
Positive for either HPV16, HPV18 or both[e]	55	6.4	4.9-8.3	23	5.3	3.4-7.8	10	5.8	2.8-10.4
Positive for any of the 12 non-vaccine HR HPV types[e,f]	233	27.1	24.2-30.2	104	23.8	19.9-28.1	50	28.9	22.3-36.3
HPV-negative	591	68.8	65.6-71.9	320	73.2	68.8-77.3	116	67.1	59.5-74
Total	859	NA		437	NA		173	NA	

CI: confidence interval; HPV: human papillomavirus; HR: high-risk; NA: not applicable.

[a] Any first cervical cell sample, independent of date.

[b] Any second cervical cell sample, at least 3 months after the first cell sample.

[c] Any third cervical cell sample, at least 3 months after the second cell sample.

[d] At least one of the HR HPV types: 16, 18, 31, 33, 35, 39, 45, 51, 52, 56, 58, 59, 66 and 68.

[e] Results are overlapping, as a sample can be positive for both HPV16/18 and non-vaccine HR HPV types due to coinfections.

[f] At least one of the non-vaccine HR HPV types: 31, 33, 35, 39, 45, 51, 52, 56, 58, 59, 66 and 68.

Supplementary Table S7. Persistent and incident HPV infections in the total of retested vaccinated (n = 7800) and unvaccinated (n = 859) women between first and second, and between second and third Trial23 cell samples, Denmark, 1 February 2017–29 February 2024

Vaccinated women				
HPV type	Consecutive sample			
HPV16/18				
**Cell sample 1**	**Cell sample 2**			**Total**
	**HPV16/18**	**No HPV16/18**	**No test**	
HPV16/18	8	12	8	28
No HPV16/18	6	5372	2394	7772
Total	14	5384	2402	7800
Persistence (HPV16/18)	45%			
Incidence (HPV16/18)	0.2%			
Numbers too small to report data for second vs. third cell sample				
Non-vaccine HR HPV types				
**Cell sample 1**	**Cell sample 2**			**Total**
	**Non-vaccine HR HPV**	**No non-vaccine HR HPV**	**No test**	
Non-vaccine HR HPV	1017	921	566	2504
No non-vaccine HR HPV	469	2991	1836	5296
Total	1486	3912	2402	7800
Persistence (HPV other)	53%			
Incidence (HPV other)	14%			
**Cell sample 2**	**Cell sample 3**			**Total**
	**Non-vaccine HR HPV**	**No non-vaccine HR HPV**	**No test**	
Non-vaccine HR HPV	554	383	549	1486
No non-vaccine HR HPV	141	1210	2561	3912
Total	695	1593	3110	5398
Persistence (HPV other)	59%			
Incidence (HPV other)	10%			

Unvaccinated women				
HPV type	Consecutive sample			
HPV16/18				
**Cell sample 1**	**Cell sample 2**			**Total**
	**HPV16/18**	**No HPV16/18**	**No test**	
HPV16/18	17	18	20	55
No HPV16/18	6	396	402	804
Total	23	414	422	859
Persistence (HPV16/18)	49%			
Incidence (HPV16/18)	1.5%			
Numbers too small to report data for second vs. third cell sample				
Non-vaccine HR HPV types				

Cell sample 1	Cell sample 2			Total
	Non-vaccine HR HPV	No non-vaccine HR HPV	No test	
Non-vaccine HR HPV	80	59	94	233
No non-vaccine HR HPV	24	274	328	626
Total	104	333	422	859
Persistence (HPV other)	58%			
Incidence (HPV other)	8%			
**Cell sample 2**	**Cell sample 3**			**Total**
	**Non-vaccine HR HPV**	**No non-vaccine HR HPV**	**No test**	
Non-vaccine HR HPV	43	30	31	104
No non-vaccine HR HPV	7	93	233	333
Total	50	123	264	437
Persistence (HPV other)	59%			
Incidence (HPV other)	7%			

Supplementary text on comparison with other studies from the discussion on page 14-17

Several other types of cancers are known to be causally associated with infection by HR HPV types. However, in this paper, we focus on the potential impact of HPV vaccination on the future need for cervical screening; therefore, we will refer only to cervical cancer in the following discussion.

In the routine statistics on cervical screening in Denmark, coverage is calculated for women aged 27-29 as the percentage of women in the population register on a given date with at least one cell sample within the last 3.5 years [1]. For 2018-2022, this percentage was 72% for the Trial23 study area, thus very close to the coverage at 3 to 4 years seen in Figure 1, showing consistence between data despite different calculation methods. The number of second cell samples in our study peaked in 2021/2022 probably due to some COVID-19 related delay [2].

In our data, the prevalence of HPV16/18 in unvaccinated women was around 5%, much lower than the 17-19% reported for young Danish women in the pre-vaccination period [3], which strongly indicated herd immunity. In the School Health Survey, 24% of girls born in 1994 reported to have been sexually active at the age of 14 [4], and girls were not HPV tested prior to vaccination. We were, therefore, not able to determine whether the few HPV16/18 infections in first cell sample in vaccinated women were present already at the time of vaccination or were breakthrough infections. In any case, the high level of persistence of HPV16/18-infections with no difference between vaccinated and unvaccinated women supported the conclusion that the 4-valent HPV vaccine is not a therapeutic vaccine [5].

In Trial23 data from the first two years of data collection, the prevalence of non-vaccine HR HPV types was 34% [6], compared with 36-39% in the pre-vaccination cohorts [3, 7]. Now, when including Trial23 data from all seven years of data collection, the prevalence of non-vaccine HR HPV types in the first cell samples was 32%. This comparison with external data could point to the presence of cross-protection, but the cohorts were not exactly comparable.

Cross-protection was not indicated in the internal comparison between vaccinated and unvaccinated women. Across age groups, the prevalence of non-vaccine HR HPV types was slightly higher in vaccinated than in unvaccinated women, see Supplementary Figure S3. Furthermore, the incidence of non-vaccine HR HPV infections was significantly higher in vaccinated than in unvaccinated women, though at a low level. These observations could point to type replacement or unmasking [5], or it could indicate that vaccinated women were more sexually active than unvaccinated women.

In Australia, HPV vaccination with 4-valent vaccine started in 2007 for women aged 12-26 [8]. Primary HPV testing data have been reported from 2017-2019. In the 117,506 women below age 25 with at least one cell sample; 1.5% were HPV16/18-positive, and 30.9% were positive for non-vaccine HR HPV (Tables A4 and A6 in reference 29). Given the lower vaccination coverage in Australia than in Denmark, these numbers corresponded well to our findings [9]. Furthermore, the prevalence of HPV 6, 11, 16 and 18 after implementation of vaccination was lower in unvaccinated women than in women from before vaccination, supporting herd immunity [10].

Women vaccinated with the 2-valent vaccine entered the English primary HPV screening pilot from 2015 [11]. As the vaccination coverage for women aged 24-25 years increased from 0% in 2013-2014 to 55% in 2018, the prevalence of HPV16/18 decreased from 13% to 3%, and the prevalence

of non-vaccine HR HPV types stayed at 25-27% [11]. A second English study was based on HPV testing of samples collected for Chlamydia trachomatis testing taken in 2010-2011 and 2020. For women aged 16-18, the vaccine coverage was estimated to increase from 60% to 85%, and HPV16/18-prevalence decreased from 8.2% to 0.9%. For women aged 19-21, vaccine coverage increased from 0% to 86%, and HPV16/18-prevalence decreased from 14.0% to 0.6% [12]. For all other (non 16/18) HR HPV, the prevalence decreased from 34.2% to 19.3% (small numbers, CI 14.4-25.0) in the youngest, and from 39.1% to 34.8% in the oldest age-group [12]. The HPV16/18-results corresponded well between our and the two English studies. The trends in prevalence of non-vaccine HR HPV types differed, but the representativity of the Chlamydia tests may have changed over time.

In Scotland, HPV vaccination was initiated in 2008, with the 2-valent vaccine targeting girls aged 12-13, starting with girls born in 1995. In 2008-2009, vaccination coverage was 92.4% [13]. Data have been reported from cohorts born between 1988 and 1995, from whom cytology samples had been HPV tested when the women were 20-21 years old. The HPV16/18-prevalence decreased from 30.0% for women born in 1988 to 4.5% for women born in 1995 [13]. For HPV 31,33 and 45, the prevalence decreased from 14.2% to 2.6%, and for non-16,18,31,33 and 45 HR HPV, the prevalence was 28.0% and 30.8%, respectively, odds ratio 1.14 (95% CI 0.95 - 1.39). Pre-vaccination HPV16/18-prevalence seems to have been very high in Scotland, and the decrease was marked. Furthermore, the data showed cross-protection for HPV 31, 33, and 45, but not for the much larger group of non-16,18,31,33, and 45 HR HPV. In the Trial23 data, we were not able to separate data for HPV 31,33 and 45. Nevertheless, we found the prevalence of the total group of non-vaccine HR HPV types to be marginally higher in vaccinated than in unvaccinated women, and a similar pattern was seen in the Scottish data for other (non-16, 18, 31, 33, 45) HR HPV.

References

[1] The Danish Clinical Quality Program– National Clinical Registries (RKKP). *DKLS yearly report 2023. [Dansk Kvalitetsdatabase for Livmoderhalskræftscreening - Årsrapport 2023]*. RKKPs Knowledgecenter, Department for Cancer and cancer screening, https://www.sundhed.dk/sundhedsfaglig/kvalitet/kliniske-kvalitetsdatabaser/screening/livmoderhalskraeftscreening/ (27 June 2024, accessed 24 October 2024).

[2] Nonboe MH, Napolitano G, Schroll JB, et al. Impact of COVID-19 pandemic on breast and cervical cancer screening in Denmark: A register-based study. *eLife* 2023; 12: e81605.

[3] Preisler S, Rebolj M, Untermann A, et al. Prevalence of Human Papillomavirus in 5,072 Consecutive Cervical SurePath Samples Evaluated with the Roche Cobas HPV Real-Time PCR Assay. *PLOS ONE*; 8. Epub ahead of print 2013. DOI: 10.1371/journal.pone.0059765.

[4] National Institution of Public Health (NIPH). *School survey 2010 [in Danish]*. 2011.

[5] Hampson IN, Oliver AW. Update on Effects of the Prophylactic HPV Vaccines on HPV Type Prevalence and Cervical Pathology. *Viruses* 2024; 16: 1245.

[6] Lynge E, Thamsborg L, Larsen LG, et al. Prevalence of high-risk human papillomavirus after HPV-vaccination in Denmark. *International Journal of Cancer* 2020; 147: 3446–3452.

[7] Kjær SK, Munk C, Junge J, et al. Carcinogenic HPV prevalence and age-specific type distribution in 40,382 women with normal cervical cytology, ASCUS/LSIL, HSIL, or cervical cancer: What is the potential for prevention? *Cancer Causes and Control* 2014; 25: 179–189.

[8] Smith MA, Sherrah M, Sultana F, et al. National experience in the first two years of primary human papillomavirus (HPV) cervical screening in an HPV vaccinated population in Australia: observational study. *BMJ* 2022; e068582.

[9] Australia C. HPV vaccination uptake. *National Cancer Control Indicators*, https://ncci.canceraustralia.gov.au/prevention/hpv-vaccination-uptake/hpv-vaccination-uptake (2015, accessed 15 November 2022).

[10] Tabrizi SN, Brotherton JML, Kaldor JM, et al. Assessment of herd immunity and cross-protection after a human papillomavirus vaccination programme in Australia: a repeat cross-sectional study. *The Lancet Infectious Diseases* 2014; 14: 958–966.

[11] Rebolj M, Pesola F, Mathews C, et al. The impact of catch-up bivalent human papillomavirus vaccination on cervical screening outcomes: an observational study from the English HPV primary screening pilot. *Br J Cancer* 2022; 127: 278–287.

[12] Checchi M, Mesher D, Panwar K, et al. The impact of over ten years of HPV vaccination in England: Surveillance of type-specific HPV in young sexually active females. *Vaccine* 2023; 41: 6734–6744.

[13] Kavanagh K, Pollock KG, Cuschieri K, et al. Changes in the prevalence of human papillomavirus following a national bivalent human papillomavirus vaccination programme in Scotland: a 7-year cross-sectional study. *The Lancet Infectious Diseases* 2017; 17: 1293–1302.

Supplementary Figure S1. Number of women (n = 17,252) and number of cell samples (n = 18,972) examined by participating pathology departments in Trial23, 1 February 2017-29 February 2024

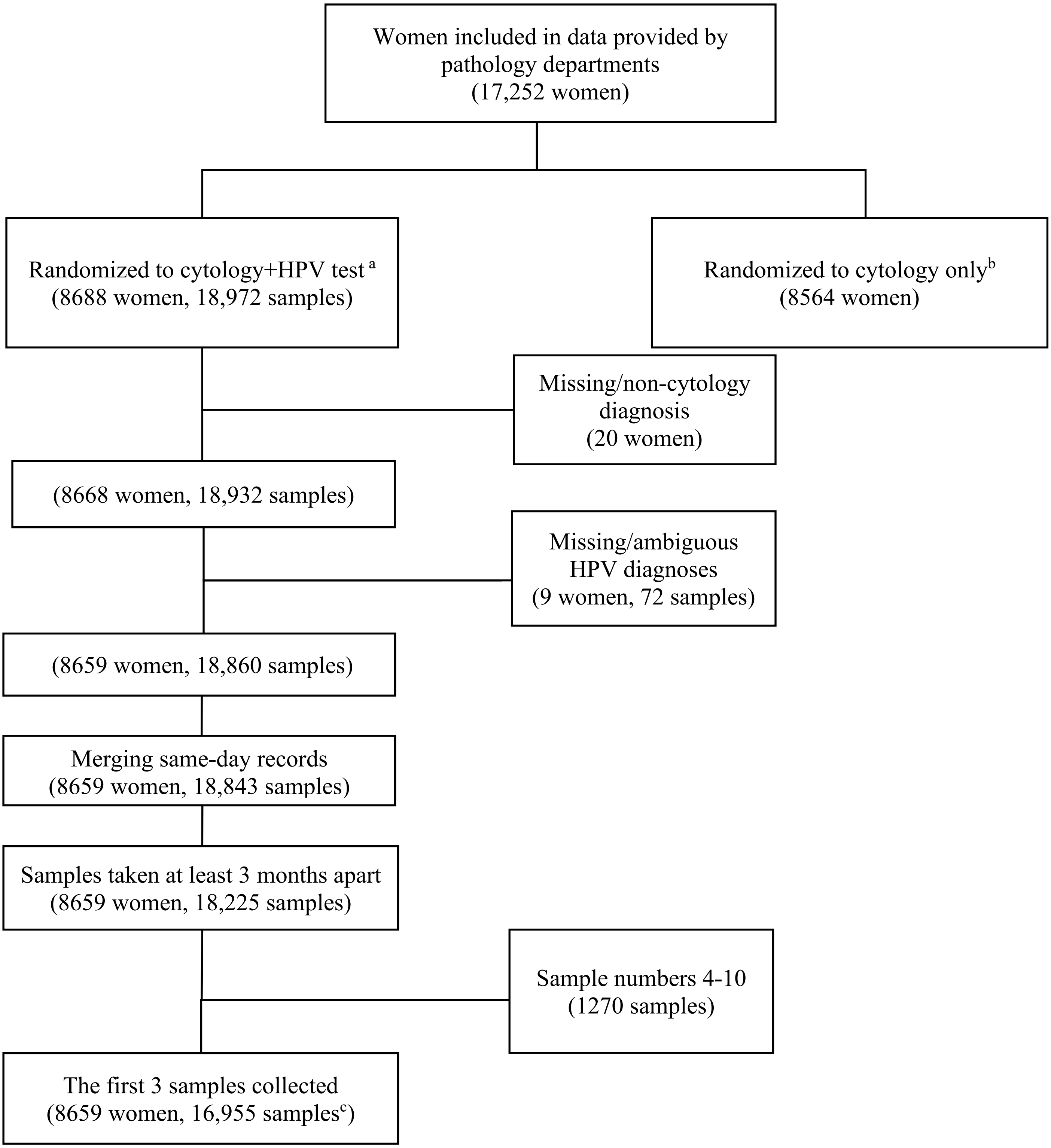

[a] Women in the Trial23 data set with at least one T23J/23J/t23j code in one sample
[b] All other women in the Trial23 data set with at least one sample (i.e. no T23J/23J/t23j)

Supplementary Figure S2a. HPV-infection flowcharts. Negative HPV-infection at first cell sample

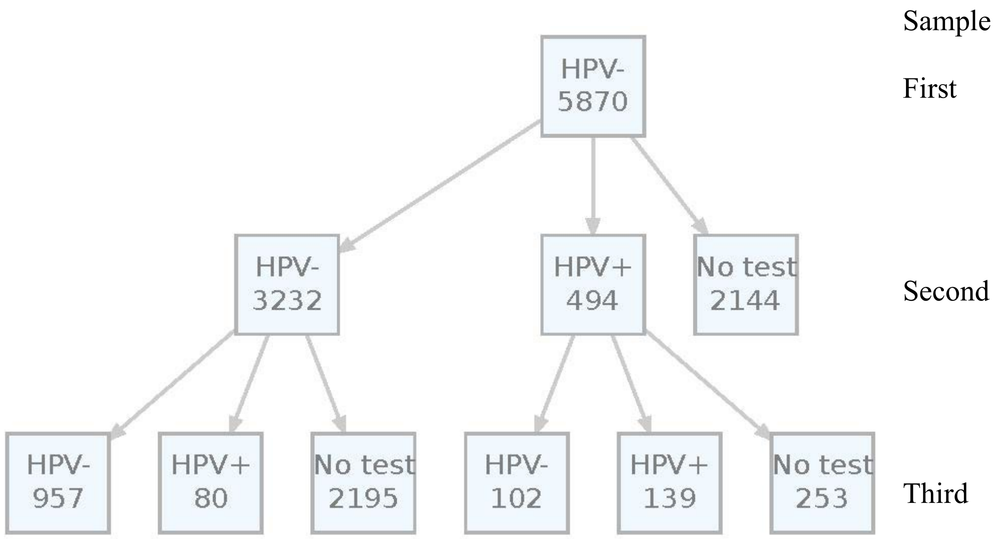

* Due to too small numbers, all HPV-types are pooled together.

Supplementary Figure S2b. HPV infection flowcharts. Positive HPV infection at first cell sample

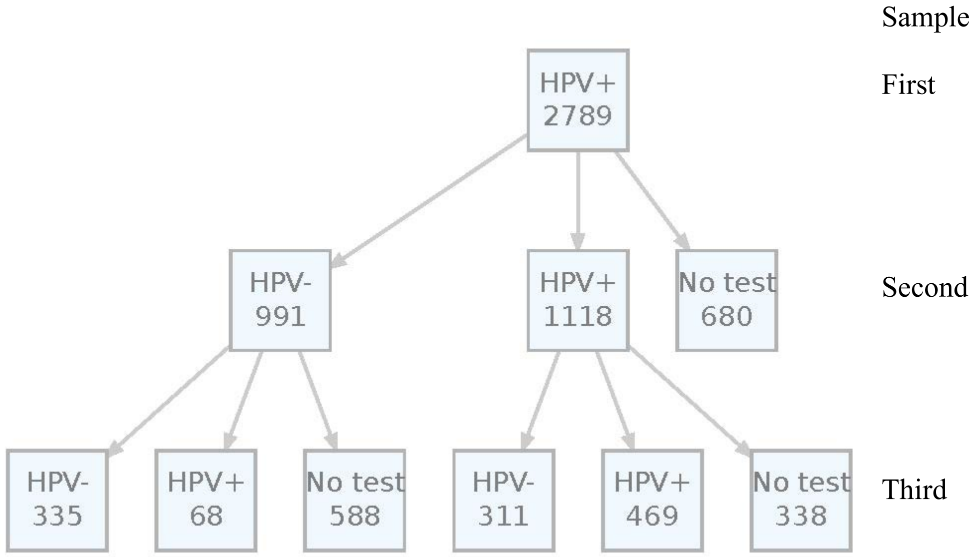

* Due to too small numbers, all HPV types are pooled together

Supplementary Figure S3. HPV prevalence by age groups

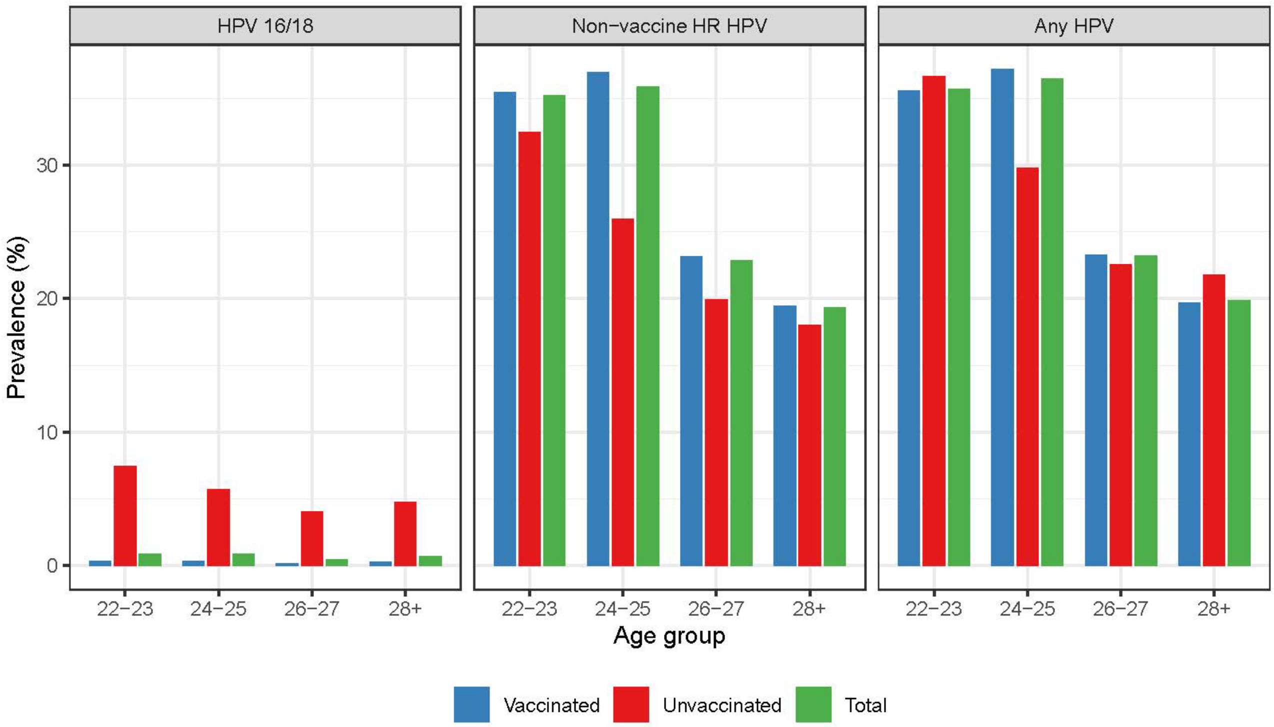

Note:
Dataset for plot was constructed by selecting out of all samples the first sample per women per age-group, disregarding the 3-months apart rule used in the main analysis.
